# Evaluation of a stepped, fixed-height magnification marker stand for use with a 100-mm marker in implant sizing prediction in 52 total hip replacement procedures in dogs

**DOI:** 10.1371/journal.pone.0280334

**Published:** 2023-01-10

**Authors:** Heather K. Siemon, William D. Liska, Sarah K. Israel

**Affiliations:** 1 Department of Surgery, Bluepearl Veterinary Partners, Tampa, Florida, United States of America; 2 Global Veterinary Specialists PLLC, Sugar Land, Texas, United States of America; 3 Department of Surgery, Bluepearl Veterinary Partners, San Antonio, Texas, United States of America; Nihon University, JAPAN

## Abstract

**Objective:**

To describe the use of a 100-millimeter marker placed on a stepped, fixed-height magnification marker stand to measure radiographic magnification on accuracy of implant size prediction when used for canine total hip replacement (THR) implant size selection.

**Study design:**

Retrospective study.

**Animals:**

Fifty-two hips in 45 dogs.

**Methods:**

This study evaluated 52 consecutive canine total hip replacement surgery pre-planning procedures involving 45 dogs with 7 undergoing staged bilateral THRs. Data collected included demographic information, measured radiographic magnification for magnification recalibration, implant size prediction of the cups (52) and the stems (52) based on digital templates superimposed on digital radiographs, and the actual implant sizes used during surgery.

**Results:**

Use of the magnification marker stand (MMS) and template application system resulted in an accurate prediction of implant size of 98/104 implants (94.2%) implants.

**Conclusion:**

A 100-mm marker placed on a magnification marker stand was a viable method to measure and recalibrate for magnification on digital radiographs during the template process to predict the THR implant sizes that should be available when the surgery begins.

**Clinical significance:**

This stepped calibration marker stand is helpful in determination of an accurate preoperative THR implant size prediction, lowering implant stock thresholds, operating time, and associated complications. Additionally, the radiographic documentation of the marker’s step height allows for indefinite confirmation of the magnification marker height used and for accurate repeatability for all follow-up imaging examinations and contralateral procedure planning.

## Introduction

Total hip replacement (THR) is often indicated for non-septic coxofemoral osteoarthritis and other trauma-induced pathology in dogs [1]. While outcomes of THR procedures are known to be good [2], implant-related complications can be devastating to the animal’s prognosis. Known implant-related complications in THR may include implant loosening, periprosthetic infection, implant failure, bearing surface wear, and femoral fracture [3].

Reports from human and veterinary medical literature have found that accurate implant sizing is related to lowering incidence of implant-related complications, and accurate preoperative radiographic planning using templating software significantly improves accuracy [4], in implant-sizing as well as lowering operative time [5]. Additionally, surgical site infection rates increase with prolonged anesthetic and surgical operative time, which suggests the need for accurate preoperative planning when possible [6].

Preoperative THR templating relies on accurate patient positioning along with appropriate magnification marker positioning (near to the relevant anatomy and parallel to the table) and calibration of magnification in the digital radiography software to account for anatomic dimensions. To calibrate for magnification, various markers, including a 100-mm stationary bar and a 25-mm spherical ball on a malleable arm have been anecdotally used in veterinary practice. However, these methods are not fool-proof. A report on magnification marker (MM) uses in human THR planning found that spherical markers were the least accurate method tested, in part because of beam divergence changing the sphere into an ellipse as well as the possibility that the ball will change position after positioned [7]. The most accurate method described in that report was case-specific measurement of the distance of the anatomy from the capture plate or film. This known distance was used to manually account for magnification on the planning images.

While preoperative radiographic planning is imperative in THR cases, there will be unavoidable deviances from the expected implant sizes due to intraoperative complexities encountered or technical challenges and the presence of degenerative changes to the anatomy in some cases [8]. In reports from human medicine, preoperative implant-size planning was considered accurate if both the templated femoral and acetabular implants were within 2 sizes of the implanted components [7, 9]. With the goal of combining the accuracy of case-specific measurement, need for lasting documentation of marker-plate distance [9], and user-friendly nature of magnification markers in preoperative THR planning, a stepped, fixed-height magnification marker stand was introduced to the veterinary market to be used with a standard 100-mm magnification marker. The stand allows the surgeon to place the marker at a known height that is captured on the image and added to the medical record so manual, case-specific measurements can be made when the planning results appear suspect.

The objective of our study is to describe a stepped, fixed-height magnification marker stand for use when capturing preoperative radiographic images of dogs receiving total hip replacement procedures. We hypothesize that the use of the magnification marker stand in conjunction with a 100-mm magnification marker will allow the surgeon to accurately predict the femoral and acetabular implant components within 1 biological fixation (BFX) implant size.

## Materials and methods

This study reviewed clinical data from medical records of dogs with complete medical records undergoing biological fixation THR between 2016 and 2018 at a single hospital. As this study is a retrospective evaluation of completed medical records, an animal ethics committee was not consulted in this research. In dogs undergoing staged, bilateral THR, each hip was included as a separate THR surgical procedure. All THR procedures included in this study used implants intended for biological fixation (BFX) from a single manufacturer (BioMedtrix LLC, Whippany, NJ). Total hip replacement procedures using other types of implants were excluded.

Information retrieved from the medical records included the signalment, body weight, and body condition score. Information retrieved from the preoperative template processes included the radiographic magnification calculated for that image and the step height on which the marker was placed. The preoperative implant plan for all procedures included a primary prediction option for the cup, stem, and all possible corresponding head sizes to be available in the operating room based on the template process measurements. In most cases, an alternative implant size option was also identified and recorded. All options were included for evaluation in this study.

### Preoperative templating

**Fig 1:**
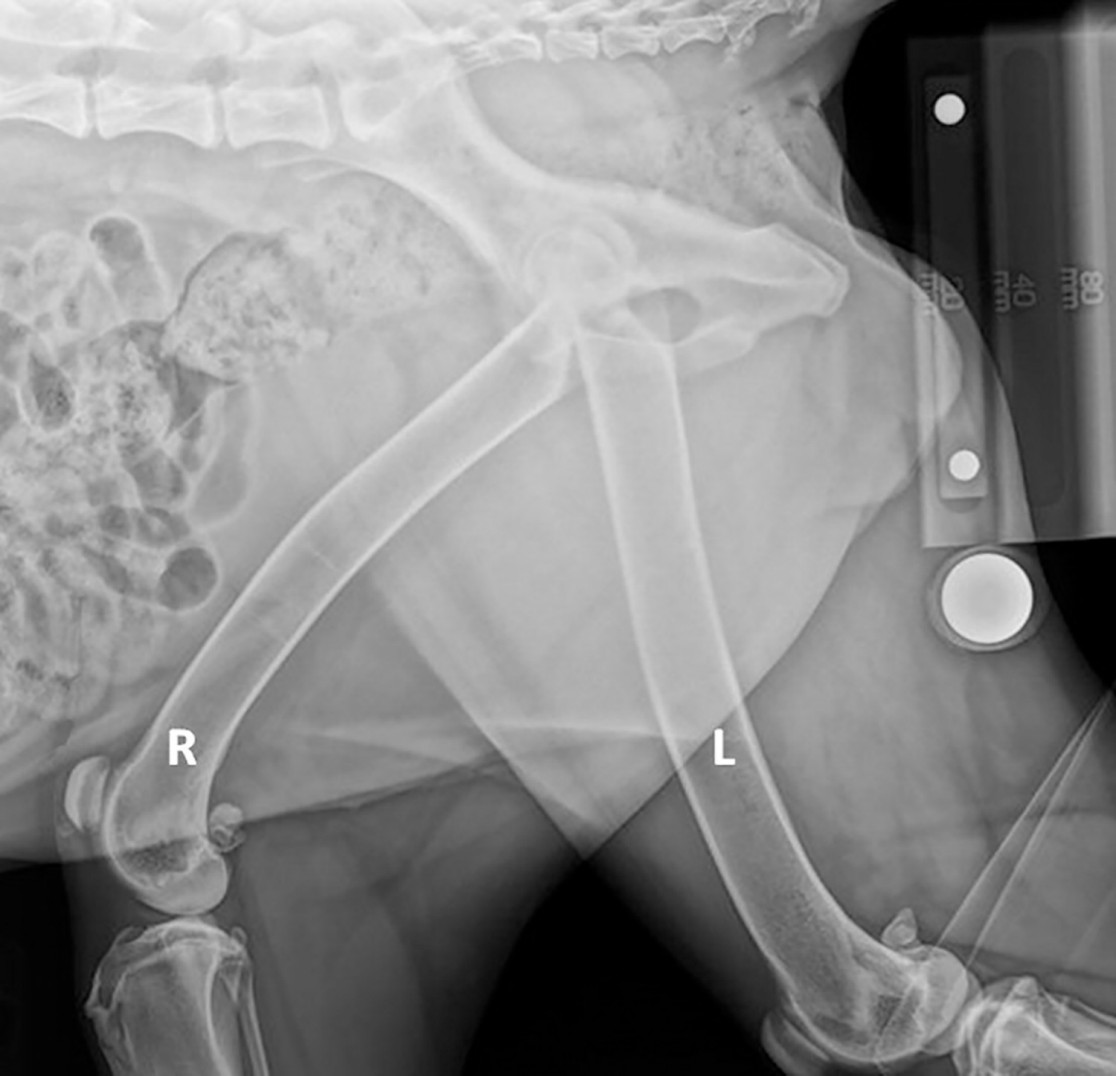
Example lateral radiograph. Lateral radiograph showing placement of the magnification marker stand with the 100 mm magnification marker placed on the 20 mm step. A 25 mm ball marker (round white object) is adjacent to the MMS for a relative comparison. Note that the left femur diameter is magnified compared to the right side. This is because the right downside was closer to the receiver. The right proximal femur is near center beam to minimize the parallax effect.

Two radiographic views of the pelvis were acquired in every dog, with additional views as needed. The views included the entirety of the pelvis and femora. A ventro-dorsal (VD) view of the pelvis was obtained with obturator foramina and ilium wings symmetrical, the hips in full extension, the femora parallel to the radiography tabletop and to one another, and the patellae centered in the trochlear grooves. A lateral view of the pelvis was acquired with the hemipelvises superimposed. The femora were not superimposed and were diverging at a 30 to 60-degree angle, with both aspects of the femoral condyles superimposed on the surgery side (Fig 1). If the femoral diaphysis was unable to be extended due to periarticular fibrosis and osteoarthritis, an additional craniocaudal view of the femur was obtained by raising the torso and tilting the pelvis to allow for a true cranio-caudal view of the femur without fore-shortening.

The surgeon requested the height (mm) at which the MM was to be placed on the MMS using palpable landmarks. To determine the height, the palpable anatomic landmark for the femoral head/acetabulum height was the greater trochanter for the VD view. The femoral diaphysis was palpated on the downside for the lateral view height. In the VD view, the MMS with the MM was abutted to the skin adjacent to the greater trochanter. The MMS and MM were placed caudal and parallel to the unaffected femur on the lateral view.

The same digital radiography software and templates (eFilm 3.3, a Sound-Eklin VCA Antech company, Carlsbad, CA) were used for all dogs in this study. This software includes a magnification recalibration tool and THR implant digital templates that superimpose over the acetabulum and proximal femur images.

**Fig 2:**
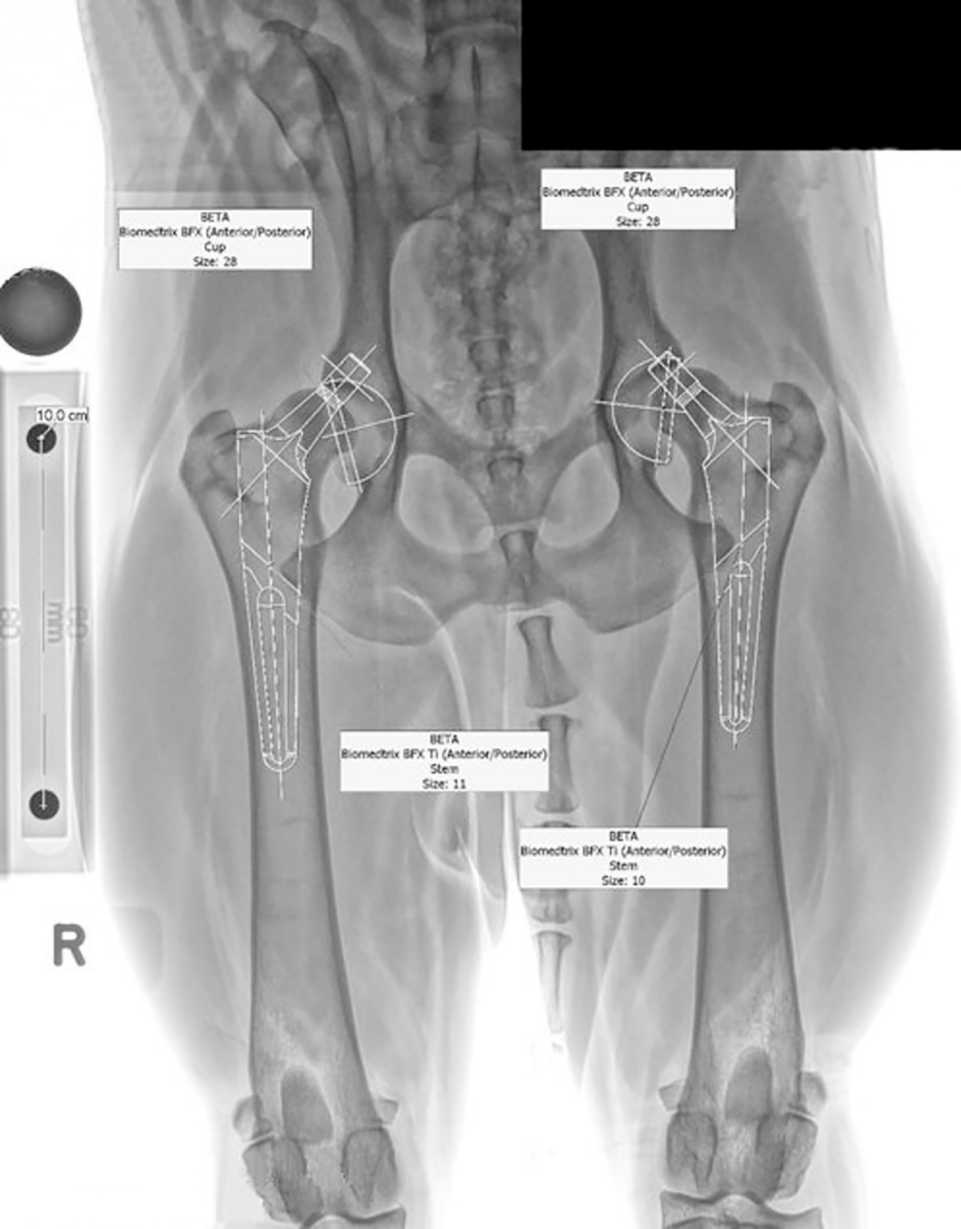
Example ventrodorsal radiograph. Ventrodorsal radiograph with the magnification marker stand and the 100 mm magnification marker placed on the 60 mm step, with a portion of the marker stand cropped out. The radiography software (eFilm 3.3, a Sound-Eklin VCA Antech Company, Carlsbad, CA) was used to recalibrate for magnification to 10.0 cm and to superimpose the implant cup and stem templates with their identity tags. A 25 mm diameter ball marker (round dark object) was sitting tabletop near the end of the MMS with the 100 mm MM for comparison. The 100 mm distance reflects the distance as measured from the center of one ball bearing to the center of the other. Likewise, the distance is 100 mm from ball bearing edge to corresponding edge because both ball bearings are the same size.

The same procedure was used preoperatively in all procedures to predict implant sizes to be used. The size of the cup implant was chosen that best matched the cranial and caudal subchondral bone dimensions of the acetabulum with the apex of the cup adjacent to the medial cortical wall. The size of the femoral stem implant was chosen based on fit inside the femoral medullary canal, with special attention paid at the anticipated femoral neck cut level, the dimensions of the proximal femoral medullary canal 2–3 mm proximal to the lesser trochanter, and to the inside diameter of the femoral medullary canal at the anticipated location of the tip of the femoral stem (Fig 2). Implant size predictions were assigned with primary being the implants most likely to be used during surgery and final implant selection. Secondary prediction was an alternative implant that would be available if the primary implant prediction was not selected. Postoperatively, the size of the implants used was recorded in the medical record and operative report.

### Device design

**Fig 3:**
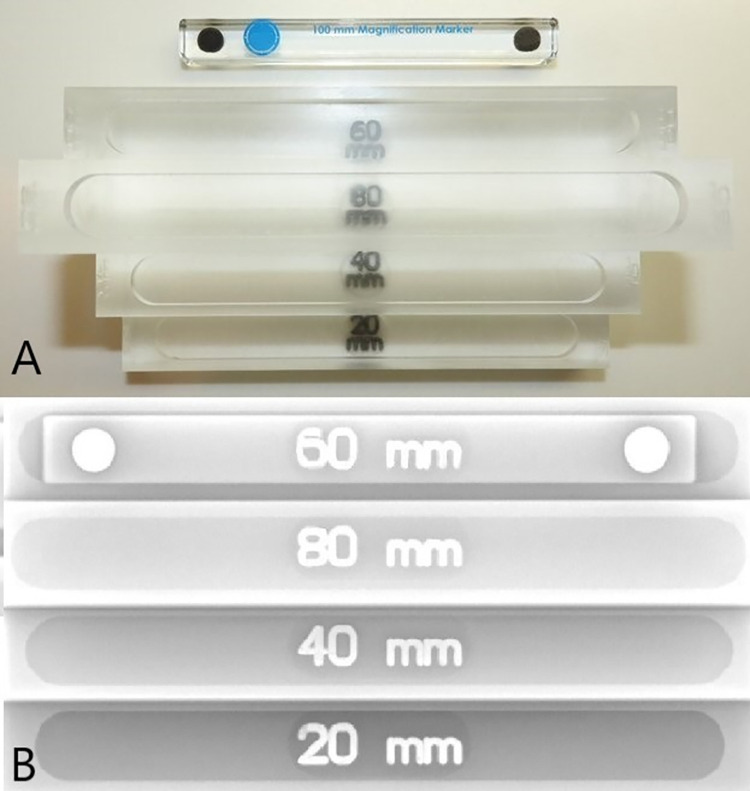
Magnification marker on magnification marker stand device. A. The magnification marker stand device with the 100 mm magnification marker adjacent to the 60 mm step. The MMS has a 20, 40, 60, and an 80 mm step for the MM to rest atop. The numbers are metallic so the height of the MM can be confirmed on the radiographs. B. A radiograph of a magnification marker stand shows the 100 mm magnification marker on the 60 mm step when the image was captured. Use of the MMS provides a permanent record of the MM height when an image is captured.

The acrylic MMS device (Global Veterinary Specialists PLLC, Sugar Land, TX) (Fig 3) has vertical steps at 20-mm, 40-mm, 60-mm, and 80-mm high. These increments are designed to hold a 100-mm linear MM placed on the desired step so that the marker is at a known vertical height above the radiography table and is parallel to the tabletop when the radiograph is acquired. Metal numbers are embedded in the MMS acrylic under each step so they appear superimposed on the MM when viewed on the radiograph. The metal numbers enable reading the MM height placement if the height chosen is not recorded. The height is indefinitely available on the radiograph image.

### Surgical procedure

The THR was performed as previously described [10] by the same 2 surgeons (SKI and WDL) in all procedures using the BFX system (BioMedtrix LLC, Whippany, NJ).

### Statistical analysis

Fisher’s exact test was used to analyze discrete data in the form of contingency tables. Statistical significance was set at P <0.05 for all analyses.

## Results

Fifty-two consecutive THR procedures met the inclusion criteria. Demographic features are summarized (Table 1). Forty-five individual dogs were included in the study with 7 staged, bilateral THR procedures. One component (either cup or stem) was not predicted accurately by the primary or secondary preoperative plans in 6/104 (5.8%) of implants used. When the primary prediction wasn’t accurate, all cases had implants that were +/-2 sizes from the primary prediction. There were no procedures in which both the cup and stem were not predicted accurately. In the 6/52 (11.5%) dogs in which one component was not predicted, 3 were cups and 3 were stems. Male and female dogs were equally represented in this study. Females were significantly less likely to have accurate implant prediction (P<0.05). In this study all male dogs had accurate implant prediction. Details about the dogs for whom an implant was not accurately predicted is provided (Table 2).

**Table 1:** Demographic information, implant size predictions, and magnification marker stand height used for dogs in this study.

**Demographics**	
Number of THR procedures	52
Number of dogs in study	45
Number of staged, bilateral THR procedures	7
Number of implants (cup, stem) predicted	104
**Age and sex**	
Female spayed	26
Female intact	0
Male castrated	22
Male intact	4
Average age (years) (range 9 months-11 years)	3.1
Average weight (kg) (range 16–54 kg)	31.16
Average BCS (out of 9)	5.6
**Breed**	
Australian shepherd	1
German shepherd	9
Golden retriever	2
Great Pyrenees	2
Labrador retriever	9
Mixed breed	15
Neapolitan mastiff	1
Pitbull terrier	3
Rottweiler	2
Shar-Pei	1
**Implants**	
Number of implants accurately predicted by either primary, secondary, or tertiary plans	94%
THR procedures where all components were predicted by plans	88%
THR procedures in which the cup was predicted by the primary plan	79%
THR procedures in which the cup was predicted by the secondary plan	15%
THR procedures in which the cup was predicted by the tertiary plan	0%
THR procedures in which the cup was not predicted in the primary or secondary plan	6%
THR procedures in which the stem was predicted by the primary plan	75%
THR procedures in which the stem was predicted by the secondary plan	17%
THR procedures in which the stem was predicted by the tertiary plan	2%
THR procedures in which the stem was not predicted in the primary or secondary plan	6%
**Marker stand height used on VD radiograph**	
20 mm	1
40 mm	15
60 mm	33
80 mm	3

**Table 2:** Information when the templating process failed to predict an implant.

Age (years)	Breed	Sex	Weight (kg)	BCS (1–9)	Measured magnification percentage	Implant predictions	Implants used	Implant not predicted	Intraoperative challenges resulting in deviation from implant prediction plan
8	German shepherd	FS	23.6	5/9	15	1^st^ 24C+8S	26C+8S	Cup	Initial caudal eccentric reaming required enlargement of the acetabular bed preparation resulting in up-size of the cup
						2^nd^ 24C+7S			
2	German shepherd	FS	39.5	7/9	14	1^st^ 24C+7S	24C+8S	Stem	The proximal femoral canal cancellous bone was soft with high porosity of the trabecular spaces in a German Shepherd. The femoral stem was upsized compared to the template prediction.
11	Labrador retriever	FS	40.0	7/9	14	1^st^ 26C+8S	26C+7S	Stem	Large medialized trochanter created challenges to implant the stem coaxially with the femoral canal; advanced proximal femoral canal sclerosis accompanying the advanced osteoarthritis resulted in down-sizing of the predicted stem.
						2^nd^ 27C+9S			
0.75	Mix	FS	20.0	4/9	10	1^st^ 22C+7S	24C+7S	Cup	A young dog with a luxoid conformation and poorly loaded acetabular soft spongy cancellous bone required an upsized cup for stable press fit fixation at the cranial and caudal poles.
						2^nd^ 6S			
1	Mix	FS	20.0	5/9	14	1^st^ 22C+6S	20C+6S	Cup	Excessive dorsal acetabular rim wear was observed on visual exposure and resulted in a decision to down-size the cup so more dorsal coverage of the cup would be present to augment cup fixation
1	Mix	FS	37.7	6/9	17	1^st^ 28C+10S	26C+9S	Stem	A mildly medialized greater trochanter shifted the femoral canal broaching medially. This factor and proximal femoral canal sclerosis accompanying the advanced osteoarthritis precluded the use of the predicted stem size, favoring down-sizing.
						2^nd^ 26C+11S			

Abbreviations: C = cup, S = stem, 1^st^ = primary plan, 2^nd^ = secondary (backup) plan

Body condition score was analyzed to determine whether dogs in high (7-9/9) or low (1-4/9) body condition scores were more or less likely to have their implants accurately predicted. No statistically significant difference (P < 0.05) was identified.

## Discussion

The preoperative template planning in all cases was able to predict implant size within 2 sizes in all cases in this study, and we accept our hypothesis. Use of the MMS with 100-mm MM resulted in accurate preoperative primary or secondary prediction of implants needed in 98/104 cases (94.2%) of the time.

Implant prediction accuracy depends on the quality and correct interpretation of radiographs with implant templates superimposed. While templating has been shown to be effective in most cases, anatomic abnormalities not identified on the preoperative imaging studies may prompt a surgeon to alter the planned implant sizes during surgery. Additionally, predicting the implant size is challenging when the anatomy for the “perfect” fit is in-between commercially available sizes. This may have been the reason why the primary prediction was not always the actual implant sized used. Of the procedures in this study, 6/104 (5.8%) implants were not accurately predicted during either the primary or secondary preoperative templating process. Due to the retrospective design of this study, we cannot identify why these decisions were made, but because of these cases, we recommend having available implants of +/- 2 sizes from the templated plan to be sure that adequate implant inventory will be available when called for intraoperatively.

All hip replacement surgeries in this study were performed by surgeons with THR experience. Experience may mitigate factors that could lead to errors for a less experienced surgeon, including those associated with accurate positioning and calibration of preoperative imaging. Additionally, seven dogs underwent staged bilateral THR. In these cases, accurate prediction of the implants needed was more likely because THR surgery was completed on the first side and the implant sizes used were known. Though this study only reflects the use of the MMS in medium and large sized dogs (Table 1), the principles of its use demonstrated in this study likely translate to use in small dogs and cats. Finally, all the dogs that failed to have their implants accurately predicted by the pre-operative templating process were female. The unexpected result is suspected to be a type two statistical error attributable to small sample size, however the possibility of significance is not excluded and warrants further investigation. A prospective follow-up study with larger cohorts is warranted to clarify these data.

**Fig 4:**
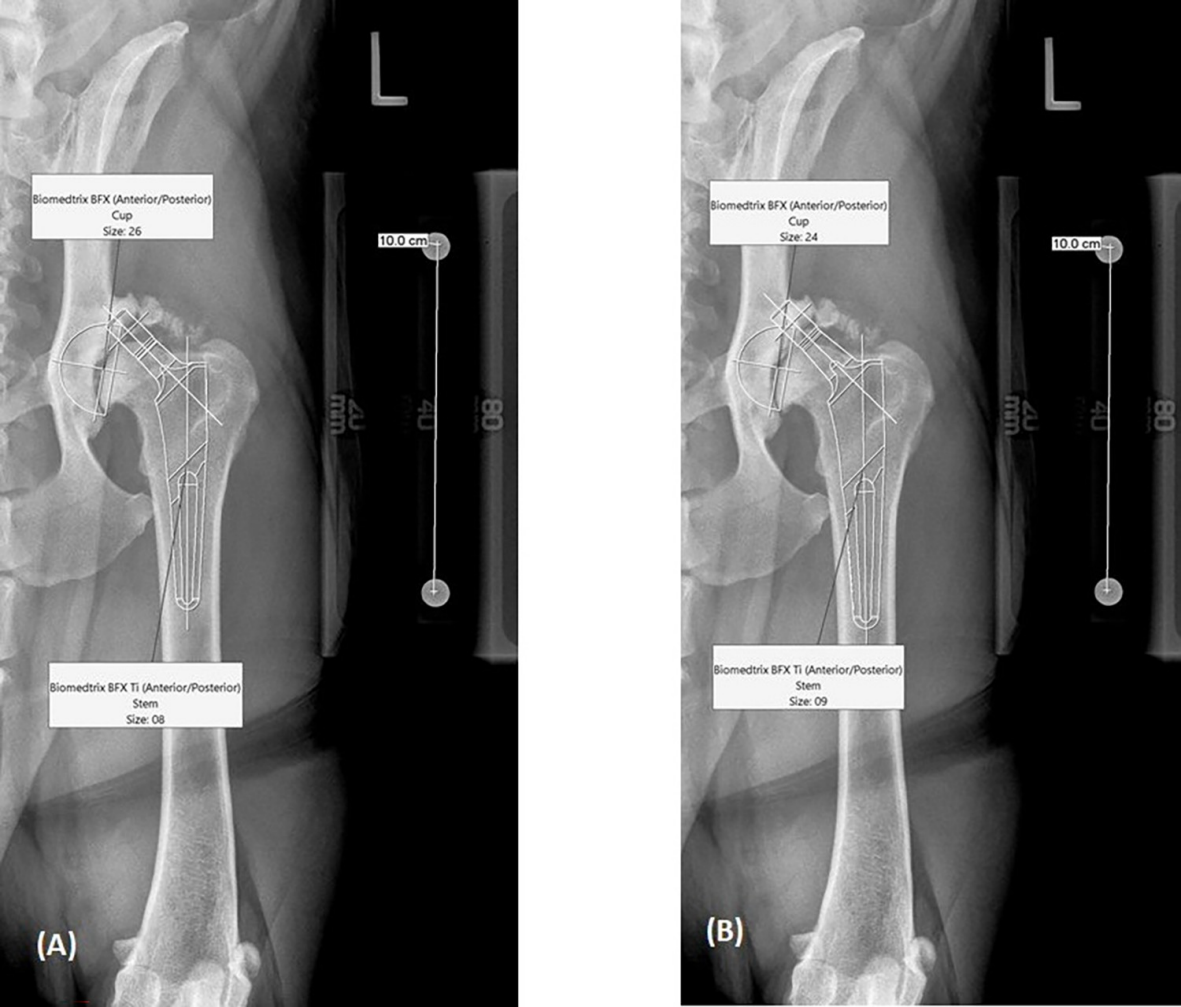
Example total hip replacement case. This case demonstrates that implant size primary prediction is not always the same as the implant selection used (secondary prediction in this case). The images are corrected for 12% magnification using the 100 mm magnification marker set on the 40 mm height above the radiology table. The cup and the stem were both in between 2 sizes in the presence of advanced osteoarthritis. Implants predicted to be used (primary plan) included a 26 mm cup and a #8 stem (A). Implants selected during surgery were a 24 mm cup and a #9 stem (secondary plan) (B). A 22 mm cup was a tertiary prediction for the worst-case scenario in anticipation of extensive acetabular filling with fibrotic tissue and bone plus extensive dorsal acetabular rim wear and flattening. If extensive intramedullary proximal femoral sclerosis would have been radiographically evident, a #7 femoral stem would have been available out of an abundance of caution.

Coxofemoral joint morphology in dogs is variable and is affected by factors such as breed, muscle mass, body conformation, physical size, and obesity. A study of human THR templating used a multiple regression model to investigate the contribution of factors such as height, weight, gender, body mass index, age, and preoperative size of the femur and acetabulum to a predictive model designed to predict THR components within +/- 1 size. Patient weight and height were found to have significant effects on the predictive model [11]. Another study found that body mass index of human patient affects some methods of radiographic scaling [7]. The contribution of the severity of osteoarthritis and physical factors, including weight, height, gender, and body mass index may also affect our ability to predict THR components in dogs (Fig 4) but has not been studied to the authors’ knowledge. A high body condition score in veterinary patients may make palpation of important landmarks difficult. Therefore, placement of marker magnification calibration devices is at an estimated height, making quantification of magnification more difficult and unreliable. We believe the reproducible height of the magnification marker on the marker stand, documented in the image, will allow for the veterinary team to capture images with the same level of magnification at all recheck exams, as well as providing training and guidance for marker height during preoperative planning of contralateral THR.

The MMS is designed so that a linear object of known length (the 100-mm MM) can be placed parallel to the X-ray receiver at a pre-determined height to facilitate more accurate radiographic magnification correction. The advantage of this device over the commonly used flexible 25.4-mm ball marker is that the height above the radiography table can be measured and requested by the surgeon or technician, the height is known precisely, and the height of the MM is permanently recorded in the radiograph image. The MMS also avoids an inherent problem associated with use of the flexible ball marker in digital radiography, for which some software programs may erroneously calibrate to 25-mm rather than 25.4-mm, or vice versa, depending on the software. Since both 25-mm and 25.4-mm ball markers are commercially available, it is imperative that the surgeon know the exact ball diameter and its digital software compatibility. In human THR planning, inappropriate positioning of calibration ball markers was shown to account for a 6.8% error in preoperative templating, and as a result of Sinclair et al.’s findings, radiographic templating using a calibration ball was recommended to only be used as a guide and not an absolute when determining correct implant size [12].

Various methods for accounting for radiographic magnification in human THR have been proposed without universal consensus. This underscores the need for improvement in calibration devices and establishing a method that is both practical and accurate. While the stepped, fixed-height magnification marker stand is a practical resource, it limits placement of the magnification marker to 20 mm height intervals, meaning that there may be up to a 10 mm variance from the correct vertical anatomical plane in a case. Multiple studies reporting on imaging magnification as relating to THR implant templating found a 1% change in magnification for every 10 mm of vertical change [13, 14]. We believe the marker stand in our study provides a method of accounting for radiographic magnification that is accurate within 1% while still being practical.

The perceived value of the surgeon being able to request the height of the MM on the MMS could not be studied because the same team of two surgeons performed all templating processes. The surgeons’ ability to train radiology technicians on how to determine the appropriate MM height on the MMS by palpation of the greater trochanter is perceived to be easy. This allows the surgeon to validate accurate MM height positioning of the technician’s estimation of greater trochanter height and vice versa. The requested height and the actual height can be verified at any time before the THR procedure starts, and any time in the future, because the height of the MM used is embedded on the radiographic image. The devices are economically feasible and training for proper use is easy.

This study was limited to a single surgery team so there are no comparisons to other surgeons’ results. The experience level of the surgeons may have mitigated the effect of any novel variable of the device proper usage. This point is reinforced by studies of human THR surgery pre-planning, which found that experience level is a significant factor in pre-operative templating predictions and implant size selection [8, 15]. A prospective study to investigate accuracy of our methods in a larger spectrum of surgeon experience would be beneficial.

## Conclusion

The magnification marker stand used with a 100-mm magnification marker is a viable method for estimating radiographic magnification during preoperative radiography to predict implants that must be available for total hip replacement surgery. Surgeons of all levels of experience may benefit from any improvement in the accuracy of their pre-surgical templating process to predict implant sizes that must be in the pre-surgery inventory and that most likely will be selected intraoperatively.

## Supporting information

S1 Data (XLSX)
